# Nitrogen fertilization compensation the weak photosynthesis of Oilseed rape (*Brassca napus* L.) under haze weather

**DOI:** 10.1038/s41598-020-60695-y

**Published:** 2020-03-04

**Authors:** Rihuan Cong, Tao Liu, Piaopiao Lu, Tao Ren, Xiaokun Li, Jianwei Lu

**Affiliations:** 1 College of Resources and Environment, Huazhong Agricultural University, Wuhan, 430070 China; 2 College of Life Sciences and Oceanography, Shenzhen University, Shenzhen, 518060 China

**Keywords:** Light stress, Environmental impact

## Abstract

Haze and cloudy weather reduce photo-synthetically active radiation (PAR), which affects the formation of crop yield and nitrogen (N) fertilizer utilization.. We conducted field trails in normal year and severe winter haze year, aiming to compare the difference of photosynthesis and N uptake in winter rapeseed under different N levels. Daily sunshine hours and averaged radiation intensity in winter haze year decreased by 54.1% and 33.3% respectively as compared with the past 30 years. Diurnal variation of net photosynthetic rate in winter haze day was 16.2% lower than that of sunny day. Leaf area and photosynthetic capacity decreased significantly during winter haze year. The shoot biomass and N uptake at the rosette stage accounted for only 9.6% and 26.6% of the total growth period in winter haze year, while 24.4% and 70.5% in normal year, respectively. However, in winter haze year, as the top dressing of N application increasing after the rosette stage, shoot biomass increased gradually. In order to achieve the target yield of 2.5 t ha^−1^, after suffering winter haze, it is necessary to apply additional 73.1 kg N ha^−1^. In conclusion, the haze climate reduced the radiation intensity and stability, leading to a decline in photosynthetic productivity in winter oilseed rape. Applying higher N fertilizer after winter haze can compensate the negative influence and ensure rapeseed yield.

## Introduction

Rapeseed (*Brassica napus* L.) is an important oil crop with an oil content of 33–50%^1,2^. In addition to extracting edible oil and feed, rapeseed can also produce margarine and artificial protein in the food industry^3^. Oilseed rape is sensitive climatic condition due to its long growing period and overwintering ability. Research in Germany found that weather conditions explained approximately 40% of rapeseed yield variability during specific growth phases^4^. In recent years, due to the increasing air pollution, haze (especially persistent haze) in the Yangtze River Basin and North China has occurred frequent in the winter season^5–7^. The Yangtze River Basin is the main producing area of winter rapeseed in China, accounting for one-fifth of cultivation acreage and rapeseed yield in the world^8^. Winter is a critical period for the growth of winter oilseed rape, and winter haze would seriously restricted the growth of rapeseed.

Several studies have reported the response of crop growth and yield to climatic parameters and have provided a biophysical basis for these factors^9–11^. Tollenaar^12^ showed that from 1984 to 2013, solar brightening contributed approximately 27% of the US Corn Belt yield trend. Simulations using a crop response model indicated that for every 1% reduction in solar brightening, crop yields are reduced by 0.7% to 1%^13^. Our previous study found that insufficient sunshine during the vegetative stage interfered with leaf photosynthesis of winter oilseed rape^9^. In addition, atmospheric aerosols and regional haze may cause a nighttime warming and a decrease in diurnal temperature range^14^, resulting in loss of crop yield^15–17^.

Nitrogen (N) is an important nutrient for plant growth. Appropriate N fertilizer supply can promote the photosynthesis and stress resistance of plants^18,19^, and improve dry matter accumulation and nutrient uptake. Winter rapeseed requires a large amount of N during the growth period, especially in the rosette stage^20,21^. Nearly 80% of N has been accumulated in the rosette stage, and the biomass accounted for 20–30%^22–24^. On the other hand, N uptake and assimilation are also affected by the environment^25,26^. Under the haze condition, the lack of photosynthetic products and reducing forces will inevitably affect the N metabolism. Studies have shown that radiation reduction leads to a significant reduction in N uptake^27,28^. Radiation reduction also has a significant impact on N utilization. It is known that under low irradiance conditions, plants invest more N in light acquisition than in light utilization, which helps the plant to maintain their state and respond to environmental changes^29–31^. Climate change will be a challenge for N fertilization^32^. The response and mechanism of dry matter production and N uptake to different N supply in winter rapeseed under the haze and cloudy conditions is unclear.

In this study, we investigated the photosynthesis and N uptake of winter rapeseed under different N fertilization rates in 2013/2014 (normal year) and 2015/2016 (severe winter haze and cloudy year), aiming to explore the difference of photosynthesis and the characteristics of N uptake under different climatic conditions. This study will help to provide stronger technical support for N management and efficient green production in winter rape production under winter sunless stress.

## Results

### Meteorological factors in the year of 2013/2014 and 2015/2016

To compare winter oilseed rape growth in the severe winter haze and cloudy year and normal year, we examined leaves area, try matter and N uptake, leaves photosynthetic parameters and climate data at rosette stage. For the whole growth period, plant samples were collected five times to analyze aboveground dry matter and N uptake. The soil was sandy loam, which was developed from granite gneiss and was classified as Ultisols. As shown in Table 1, soil pH was 5.8, and soil organic matter, total N, soil available phosphorus and available potassium were 33.1 g kg^−1^, 1.9 g kg^−1^, 6.4 mg kg^−1^ and 57.2 mg kg^−1^, respectively.

**Table 1:** Soil basic physical and chemical properties in the surface layer (0–20 cm).

pH	SOM (g kg^−1^)	Total N (g kg^−1^)	Available P (mg kg^−1^)	Available K (mg kg^−1^)	Particle size (%)^a^		
					clay	silt	sand
5.8	33.1	1.9	6.4	57.2	8.5	37.5	54.0

^a^Particle size categories: clay, <0.002 mm; silt, 0.02–0.002 mm; sand, >0.02 mm.

For the year of 2015/2016, sunshine hours and daily mean *PAR* were quite lower during the rosette stage (i.e., 69 days after transplanting), which decreased 144 h and 115 μmol m^−2^ s^−1^ with 54.1% and 33.3% decline as compared with the mean values during the same time of 1985–2014, respectively (Table 2). The average temperature and accumulated temperature had no differences with the same period of previous years, but the effective accumulated temperature (>5 °C) increased by 15 °C. For the year of 2013/2014, taking into account the normal year during the rosette stage, the number of sunshine hours and daily mean *PAR* was slightly higher than the values of the same time in the last decade.

**Table 2:** Main meteorological data within 69 days after transplantation.

Year	*T*_avg_ (°C)	*AT* (°C)	*EAT* (≥5 °C)	*SSH* (h)	*PAR* (μmol m^−2^ s^−1^)	Precipitation (mm)
2013/2014	9.3	644	618	310	368	70.5
2015/2016	9.1	629	600	122	230	126.8
1985–2014	9.1	625	585	266	345	114.2

*T*_avg_, average temperature; *AT*, accumulated temperature; *EAT*, effective accumulated temperature; *SSH*, sunshine hours; *PAR*, photosynthetically active radiation.

**Figure 1:**
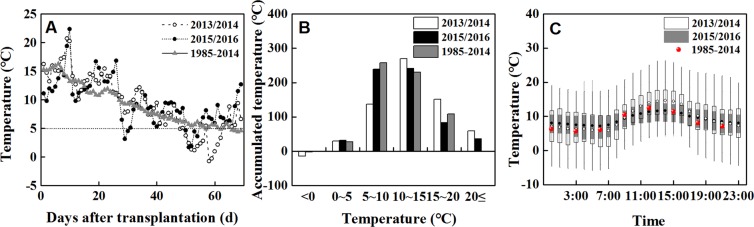
Temperature variation and distribution within 69 days after transplantation.

There were significant differences in the variation and distribution of daily temperature in 2013/2014 and 2015/2016 (Fig. 1). Daily temperature dropped to 3.1 °C in the 29^th^ day after transplanting in the year of 2015/2016 (Fig. 1A). The accumulated temperature showed a normal distribution, but its peak was smoother during winter haze (Fig. 1B). Diurnal variation of temperature showed 0.3–1.1 °C lower during 7:00–18:00 but 1.1–1.7 °C higher from 19:00 to the next 6:00 in 2015/2016 than that of 2013/2014 and the past 30 years. Clearly, haze and overcast reduced the diurnal temperature range (Fig. 1C).

**Figure 2:**
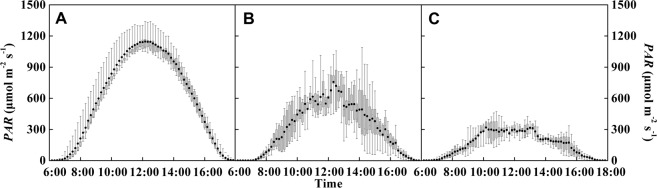
Diurnal variation of average *PAR* in sunny (**A**), haze (**B**), and cloudy-overcast (**C**) days within 69 days after transplantation in the year of 2015/2016.

In the 69 days after transplanting in 2015/2016, the diurnal variation of *PAR* on sunny days (i.e., cloud fraction is 0~0.1 and AQI <100), haze (i.e., AQI >100) and cloudy-overcast (i.e., cloud fraction is 0.1~1 and AQI <100) days were examined (Fig. 2). On sunny days, the diurnal variation of *PAR* showed a clear normal distribution with little change at each time point (Fig. 2A). However, on haze days, the change in *PAR* was disorderly, with an averaged decrease of 47.8% compared to sunny days (Fig. 2B). For cloudy-overcast days, the amplitude of *PAR* was in the smallvariation with 73.4% lower than that in sunny days (Fig. 2C).

### Morphological and physiological traits as affected by different weather conditions

**Figure 3:**
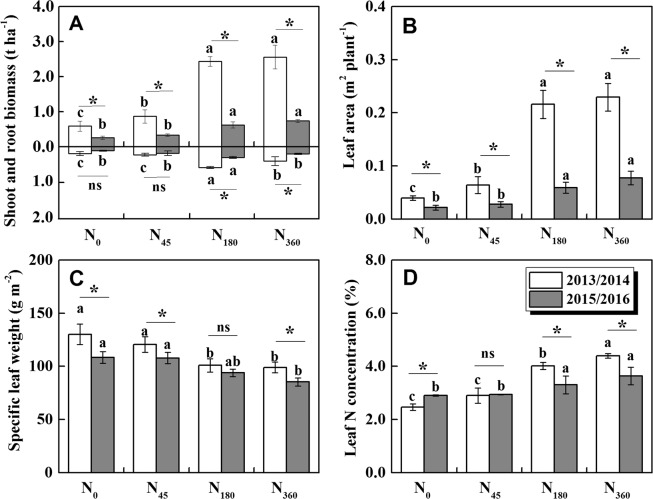
Morphological and physiological traits at the rosette stage in winter oilseed rape under N fertilizations between the years of 2013/2014 and 2015/2016. Different letters in the same year indicated significant differences among N treatments (P < 0.05). Differences between the two years in the same treatment were shown as *(P < 0.05) and ns (no significance).

N application improved leaf growthduring the rosette stage of winter rapeseed (Fig. 3). The dry matter of N_180_ treatment increased by 394.2% in shoot and 193.0% in root compared with N_0_ treatment in 2013/2014, and increased by 174.9% and 128.4% in 2015/2016, respectively (Fig. 3A). Our results revealed that winter haze and cloudy reduced the effect of N fertilizer on the accumulation of dry matter. Compared with the year of 2013/2014, the leaf area of different N treatments decreased by 45.6–76.9% in 2015/2016 (Fig. 3B). However, reductions of specific leaf weight (6.9–16.8%) and leaf N concentration (−18.0–17.3%) were smaller among the treatments between the two years (Fig. 3C,D).

### Photosynthetic ability under different weather conditions

**Figure 4:**
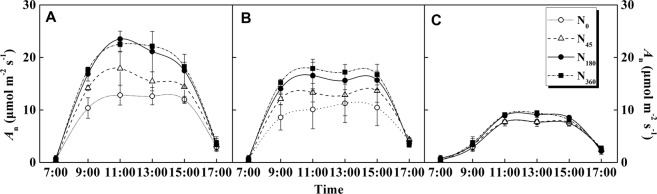
Effects of different N application rates on photosynthetic diurnal dynamics of winter oilseed rape leaves under sunny (**A**), haze (**B**) and cloudy-overcast (**C**) days in the year of 2015/2016.

Diurnal variation of photosynthetic rate under different N application rates was various in different weather conditions (Fig. 4). Net photosynthetic rate (*A*_*n*_) of sunny and haze days increased with N inputs among N application treatments, and there was no significant difference in *A*_*n*_ of cloudy-overcast days. For sunny days (Fig. 4A), daily maximum value of *A*_*n*_ was observed at 11:00, ranging from 12.8–23.5 µmol CO_2_ m^−2^ s^−1^. There was no significant difference in the *A*_*n*_ value between N_180_ and N_360_ treatments on sunny days. In case of haze days (Fig. 4B), daily maximum *A*_*n*_ in N_360_ was significantly higher than that in N_180_ treatment. However, in cloudy-overcast conditions, the diurnal variation of *A*_*n*_ was lower than in sunny and haze days, and there was little difference among the treatments (Fig. 4C). The results indicated that the promoting effects of N fertilization on the photosynthetic productivity was closely related to the radiation intensity at rosette stage of winter rapeseed.

Analysis of light response curve revealed that the net photosynthetic rate increased with the increase of the irradiation intensity, and the photosynthetic rate increased rapidly first and then stabilized (Fig. S2). N fertilization application increased the net photosynthetic rate, especially under high light intensity (PAR >500 μmol m^−2^ s^−1^). Further analysis of the characteristic parameters of the light response curves indicated that the apparent quantum efficiency (AQY), light compensation point (LCP), light saturation point (LSP) and the maximum net photosynthetic rate (*A*_*max*_) of the leaf in 2015/2016 were significantly lower than 2013/2014 (Table 3).

**Table 3:** Effects of different N application levels on parameters of light response curve of leaves in winter oilseed rape.

Year	Treatments	*AQY*	*LCP* (μmol m^−2^ s^−1^)	*LSP* (μmol m^−2^ s^−1^)	*A*_,max_ (μmol m^−2^ s^−1^)
2013/2014	N_0_	0.041 b^ns^	57.7 c*	1244 b^ns^	15.0 b*
	N_180_	0.050 a*	78.9 a*	1535 a*	18.3 a*
	N_360_	0.053 a*	67.5 b*	1583 a*	20.1 a*
2015/2016	N_0_	0.038 c	35.4 c	1150 b	10.2 c
	N_180_	0.043 b	55.2 a	1393 a	15.4 b
	N_360_	0.049 a	42.9 b	1451 a	18.6 a

Different letters in the same column at a given year indicated significant differences between N treatments (P < 0.05). Differences between two years in the same treatment are indicated: *(P < 0.05) and ns (no significance).

### Biomass and N uptake under different weather conditions

**Figure 5:**
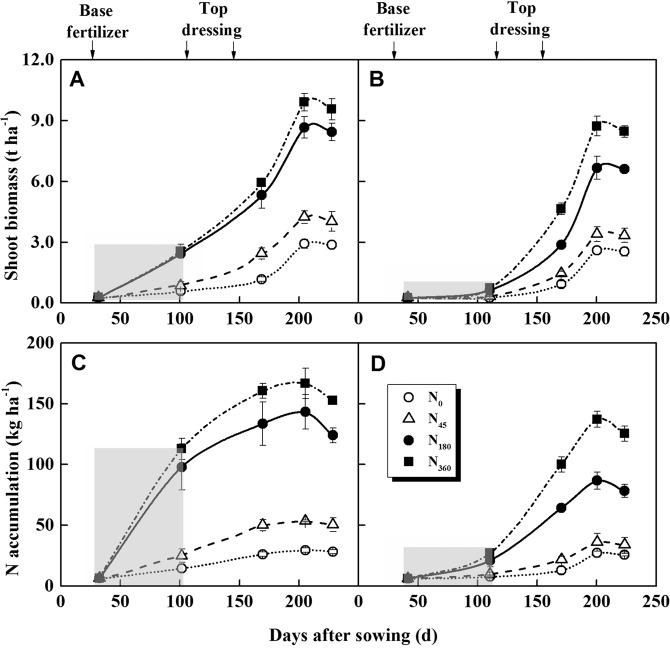
Shoot biomass and N accumulation during the growth period of oilseed rape in 2013/2014 (**A,C**) and 2015/2016 (**B,D**). Shaded area represented winter haze occurred in 2015/2016 and the same period in 2013/2014.

The haze climate had a significant effect on shoot biomass production and N uptake during winter rapeseed growth (Fig. 5). As shown in Fig. 5, the shoot biomass and N accumulation in the rosette stage only accounted for 9.6% and 26.6% of the entire growth period in the winter haze year, and normal year were 24.4% and 70.5%, respectively. However, the topdressing high N fertilizer could significantly increase the rapeseed biomass and N uptake after the winter haze. Compared with N_180_ treatment, the shoot biomass and N accumulation of N_360_ treatment increased by 28.0% and 60.1% at mature period in 2015/2016, respectively, and only increased by 13.4% and 23.3% in 2013/2014. Eventually, the shoot biomass (8.5 t ha^−1^) and N accumulation (125.1 kg N ha^−1^) of N_360_ treatment in 2015/2016 mature period were comparable to the 2013/2014 N_180_ treatment.

### Yield and N use efficiency under different weather conditions

**Figure 6:**
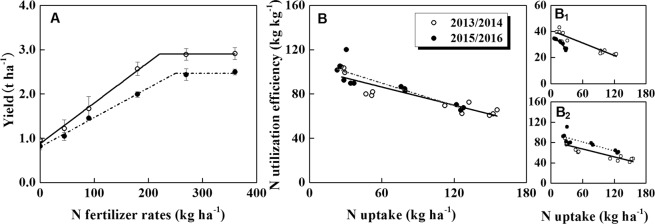
Rapeseed yield (**A**) and N utilization efficiency (**B**) in 2013/2014 and 2015/2016. B_1_ and B_2_ denote the relationship between N uptake and N utilization efficiency during rosette stage and after rosette stage, respectively.

According to the linear-plateau model (Fig. 6A), the appropriate N application rates were 220.1 kg ha^−1^ in the year of 2013/2014 and 249.7 kg ha^−1^ in the year of 2015/2016, with the plateau yields of 2.9 t ha^−1^ and 2.5 t ha^−1^, respectively. Compared with the normal weather, additional 73.1 kg N ha^−1^ was needed to reach a target yield of 2.5 t ha^−1^ during the haze and cloudy weather. Regardless of the haze or not, there was no significant difference in N utilization efficiency (N dry matter production efficiency) under the same N uptake (Fig. 6B). Further analysis found that in the haze and cloudy weather, the N utilization efficiency of the rosette stage was significantly lower than that of the normal weather (Fig. 6B_1_) and the N utilization efficiency of the later stage was higher than that of the normal weather (Fig. 6B_2_).

## Discussion

### Climate factors impact photosynthetic productivity

Low temperature, overcast and rainy, and sunless would reduce the light intensity, resulting in a serious lack of photosynthesis driving force, and a sharp decline in photosynthetic rate. In this study, daily photosynthetic rate of haze and cloudy-overcast days were significantly lower than that of sunny days. Haze not only reduced the light intensity, but also increased the proportion of blue and green light^33^. Green light is inefficient for photosynthesis, which would result in the decrease of photosynthesis and thus inhibiting plant growth. In addition, haze days would cause fluctuations in light intensity due to the influence of air currents on aerosols and solid particles in the air. Photosynthetic capacity in unstable light conditions was significantly lower than that of normal weather. N nutrition can improve crop’s ability of rapid response to unstable light^34,35^, indicating that with the increase of N application rate, the photosynthetic daily assimilation amount of winter rapeseed increased. In addition, the haze climate reduced the daily temperature range and increased the nighttime temperature^14^, which is consistent with the results of this study. The higher nighttime temperature is not conducive to dry matter accumulation of plants^15–17^.

### N fertilization after haze: a compensation strategy to ensure crop yield

Plants often showed strong plasticity, and in adversity there are always multiple regulatory mechanisms to maintain their growth. In addition, N nutrition can increase the tolerance of plants to stress. Our results demonstrated that winter haze caused a significant decrease in dry matter and leaf area, but the root/shoot ratio increased significantly during the rosette stage. Previous results have shown that under the light restriction, plants allocated more resources to shoots for competing light resources^36^. However, ontogenetic, plastic, and growth limitation responses often occurred simultaneously^37^. Winter haze would severely inhibited the ontogenetic and growth of winter rape at rosette stage, thus increasing root/shoot ratio. On the other side, N nutrition could promote shoot biomass accumulation and decrease root/shoot ratio. We found that shoot biomass and N uptake were increased rapidly after winter haze, especially for N applications (Fig. 5). In recent years, the seed yield in this area was maintained at 2.6 t ha^−1^ (2.2–3.0 t ha^−1^, 75% QR) under normal conditions^38–41^, which was close to the yield of the control treatment in this study. Therefore, winter haze is the main reason for the decrease of seed yield in the year of 2015/2016.

Some studies showed that plants have a certain ability to compensate for the effects of early shading stages^42^. In our study, no significant increase in shoot biomass and N uptake was observed during the normal year when N application increased to 180 kg N ha^−1^. The effect of winter haze on N uptake in the early growth stage of winter rapeseed was significant, and there was no differences between the N_180_ and N_360_ treatments. Under the N_360_ treatment, the shoot biomass and N uptake increased significantly after the first topdressing, which indicated that increasing the input of N fertilizer after winter haze could compensate for the influence of haze. After winter haze, increasing the amount of N fertilizer can significantly improve N absorption and utilization. Therefore, rational N fertilizer application in winter haze and cloudy weather would be an effective way to reduce N loss, increase yield and N use efficiency.

## Conclusions

Winter haze and cloudy reduced the intensity and stability of irradiation, which lead to a decrease in photosynthetic productivity of winter rapeseed, and a significant reduction in leaf area and photosynthetic capacity. Increasing the amount of top dressing of N application could be benefit for plant growth after winter haze. In order to achieve the target yield of 2.5 t·ha^−1^, additional 73.1 kg N·ha^−1^ was needed in the haze year. Applying higher N fertilizer after winter haze and cloudy could compensate the negative effects and ensure rapeseed yield.

## Methods

### Experimental sites

Field experiments were conducted in Wuxue County (30 °06′N, 115 °35′E), Hubei province, in the year of 2013/2014 and 2015/2016. The site was in the subtropical monsoon climate zone. The climate is featured by distinctive seasons, humid but low light. Mean annual temperature is 16.8 °C. Mean annual precipitation is 1316 mm, with frequent rainstorms, cloud and fog. Mean annual sunshine duration is less than 1400 hours.

### Experimental design

Six N rates (i.e., 0, 45, 90, 180, 270, 360 kg N ha^−1^) were taken in the year of 2013–2014 and 2015–2016. Other fertilizers were applied at 90 kg P_2_O_5_ ha^−1^, 120 kg K_2_O ha^−1^ and 1.62 kg B ha^−1^, respectively. The fertilizers sources were urea (46% N), calcium superphosphate (12% P_2_O_5_), muriate of potash (60% K_2_O) and borax (10.8% B). For all the sites, P, K and B fertilizers were applied to the soil as basal fertilizers. N fertilizer was applied in three splits: 60% prior to transplanting, 20% at the overwintering stage (about 60 d after transplanting), and 20% at the initiation of stem elongation (about 100 d after transplanting). The experiment was designed with three replications, and the random block was arranged. Plot size was 20 m^2^ for each replication.

Local commercially cultivar of Huayouza No. 9 was used in this study. Rapeseeds were firstly sown in prepared seedbed on Sep 27^th^ in the year of 2013/2014 and Sep 24^th^ in the year of 2015/2016. To ensure the similar size of basic seedlings for the 2013/2014 and 2015/2016, plant with 4 leaves (about 35 d after sowing) was transplanted into the tilled field. The plant density was 11.25 × 10^4^ plants ha^−1^ in each experiment. Field management were performed following local methods, such as herbicide application and pest and disease controls. No obvious weed, pest, or disease stresses were observed during the rapeseed cropping seasons of the two years.

### Sampling and measurement

Composite soil samples (10 cores per site) were collected from the top 20 cm of the soil profile before the experiment. The samples were air dried and crushed to pass through 1 mm sieve for a chemical analysis. Soil pH (1:2.5 soil water ratio), organic C (dichromate oxidation method), total N (Kjeldahl acid-digestion method), and Olsen-P, NH_4_OAc-K were also measured.

Plant samples were collected five times, i.e., the seedling (GS1.0, which used as basic seedlings for transplanting), rosette (GS2.2), flowering (GS4.1), pod (GS5.1), and maturity (GS5.5) stages^43^. Six plant samples (shoot and root) of four N treatments (0, 45, 180 and 360 kg N ha^−1^) were randomly collected to determine dry matter and N uptake. The leaves of rosette (GS2.2) stage were placed on black cardboard and 5 × 5 cm green cardboard was added as a control. The leaf area was obtained by using a digital camera (D700, Nikon, Inc. Japan) and image-pro plus 6.0 software^44^. Subsequently leaf dry matter and total dry matter were determined by drying at 105 °C for 30 min and oven-drying at 60 °C to constant weight^45^. All dried and milled plant fractions were digested with H_2_SO_4_-H_2_O_2_^46^. The N concentration in the digestion solution was analyzed by the Continuous Flow Analysis (AA3, Seal Analytical Inc., Southampton, UK). Rapeseed yield was determined by a harvest area of 10 m^2^ for each plot at mature. The moisture content of seeds at harvest was 8% to 12%, and the yield was expressed on the basis of dry matter.

### Determination of photosynthetic parameters

Photosynthetic light response curves and diurnal photosynthetic rate of newly expanded leaves were measured by Li-Cor 6400 XT (Li-Cor, Lincoln, NE, USA) portable photosynthesis open system. From 9: 00 to 15: 30, light response curve was measured at rosette (GS2.2) stages. The equipment maintained the relative humidity of the air in the leaf chamber at 50–70%, and the leaf temperature at 20 °C. The light intensive sequence was controlled be an automatic program, with 120 s response time for each test point. The photosynthetic photon flux density (PPFD) was controlled across a series of 1800, 1500, 1200, 1000, 800, 600, 400, 200, 100, 50, 0 μmol m^−2^ s^−1^. The diurnal photosynthetic rate was measured every 2 h from 7:00 to 17:00, and the natural conditions were determined. The reciprocal measurement was adopted to eliminate the errors of the measurement time.

According to Thornley^47^ and linear function model, the parameters of photosynthetic light response curve were estimated by non-rectangular hyperbola function. The simulation equation was:

1 $${A}_{{\rm{n}}}=\frac{\varphi {\rm{PPFD}}+{A}_{{\rm{\max }}}-\sqrt{{(\varphi {\rm{PPFD}}+{A}_{{\rm{\max }}})}^{2}-4{k}{\varphi }{\rm{PPFD}}{A}_{{\rm{\max }}}}}{2k}-{R}_{{\rm{d}}}$$

where *A*_n_ was the net photosynthetic rate; *φ* was the apparent quantum efficiency (AQY); PPFD was photosynthetic photon flux density; *A*_max_ was the maximum net photosynthetic rate at the light saturation point; *k* was the curvature parameter; *R*_d_ was the dark respiration rate. AQY was estimated as the slope of the photosynthetic when PPFD was below 200 μmol m^−2^ s^−1^. The light compensation point (LCP) was estimated from the x-axis intercept, and the light saturation point (LSP) was estimated from the intersection with *A*_max_.

### Meteorological data

Meteorological data were collected by the Automatic Meteorological Station (AWS 800, Campbell Scientific, Inc., America). The main observation items included solar radiation, atmospheric temperature, rainfall and photosynthetically active radiation (*PAR*). The measurement time step was 10 min. Air Quality Index (AQI) data were obtained from China National Environmental Monitoring Centre, including PM2.5, PM10, SO_2_, NO, O_3_, CO, etc. The measurement time step was 1 h. Cloud fraction was available from both the Terra (MOD06) and Aqua (MYD06) satellites and can be surpassed during the day and night (https://worldview.earthdata.nasa.gov/). The sensor/algorithm resolution was 5 km, the image resolution was 2 km, and the temporal resolution was daily.

For this study, the weather conditions within 69 days after transplantation (winter haze occurred) were categorized into three types, i.e., sunny (cloud fraction is 0~0.1 and AQI <100), cloudy and overcast (cloud fraction is 0.1~1 and AQI <100) and haze (AQI >100). According to the air quality index (AQI) and cloud fraction (Fig. S1), the 69 days after transplanted were divided into different weather conditions. There were 32 sunny days, 10 haze days and 27 cloudy-overcast days in the year of 2013/2014. For the year of 2015/2016, there were only 8 sunny days but 30 haze days and 31 cloudy-overcast days.

### Data analysis

ANOVA and least-significant-difference (LSD, *P* = 0.05) were applied to compare measured parameters among the treatments using SPSS 18.0 (SPSS Inc., Chicago, IL, USA). To calculate the optimum N rate for each experiment, linear-plateau model was used to fit the rapeseed yield under different N application levels, which would reflect yield response to fertilization effectively^48^. Linear-plateau regression models was fit using PROC REG program (SAS Institute, Cary, NC).

The dry matter production per unit N was used as N utilization efficiency (NU_t_E). NU_t_E was were calculated as

2 $${{\rm{NU}}}_{{\rm{t}}}{\rm{E}}={{\rm{B}}}_{{\rm{up}}}/{{\rm{N}}}_{{\rm{up}}}$$

where N_up_ represented the total N uptake in shoot; B_up_ was the biomass of shoot.

## Supplementary information

Supplementary materials.
