# Supplementary material for: Nitrogen fertilization compensation the weak photosynthesis of Oilseed rape (Brassca napus L.) under haze weather
Source: Sci Rep. 2020 Mar 4;10:4047. doi: 10.1038/s41598-020-60695-y (PMC7055290; doi:10.1038/s41598-020-60695-y)
Supplement: Supplementary file 1 — Supplementary materials. [file 41598_2020_60695_MOESM1_ESM.pdf]

Nitrogen fertilization compensation the weak photosynthesis of Oilseed rape (*Brassca napus* L.) under haze weather

Rihuan Cong [a], Tao Liu [a,b], Piaopiao Lu [a], Tao Ren [a], Xiaokun Li [a], Jianwei Lu [a *]

[a] College of Resources and Environment, Huazhong Agricultural University, Wuhan 430070, China

[b] College of Life Sciences and Oceanography, Shenzhen University, Shenzhen 518060, China

[*]**Corresponding author:**

Prof. Jianwei Lu

Tel: +86-27-87288589

Fax: +86-27-87288589

E-mail: lunm@mail.hzau.edu.cn

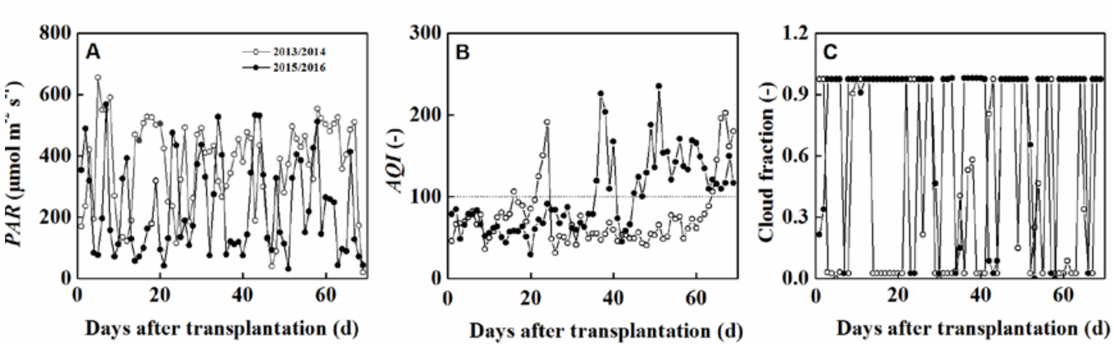

Figure S1. Daily mean PAR variation, AQI and cloud fraction within 69 days after transplantation.

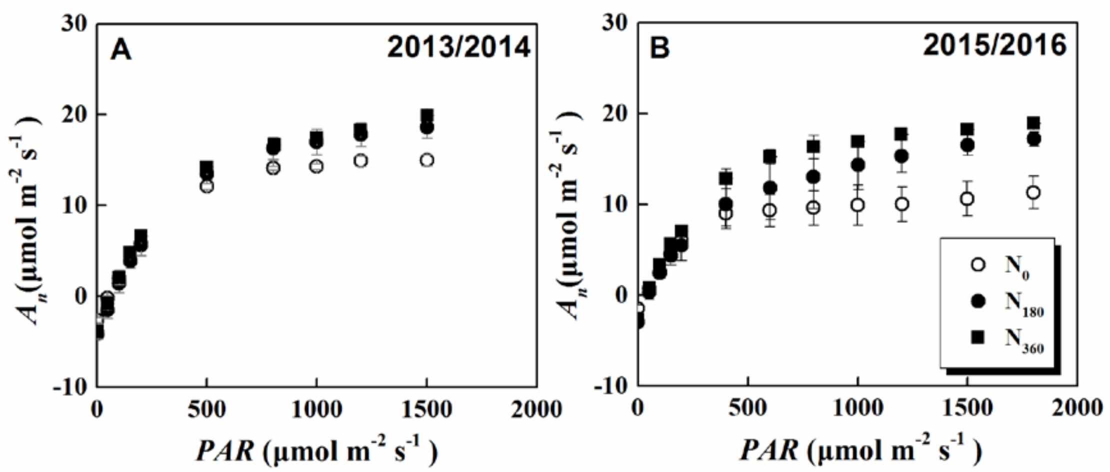

Figure S2. Effects of different nitrogen application rates on light-response curves of winter oilseed rape leaves in the season of 2013/2014 and 2015/2016.
